# Intraoral Inflammatory Leiomyosarcoma: A Case Report and Literature Review

**DOI:** 10.7759/cureus.63399

**Published:** 2024-06-28

**Authors:** Mohammed M AlAli, Abdulsalam Aodah

**Affiliations:** 1 Pathology, Riyadh Regional Lab, Riyadh, SAU; 2 Oral and Maxillofacial Surgery, King Fahad General Hospital, Hofuf, SAU

**Keywords:** sarcoma, myod1, soft tissue, intraoral, inflammatory leiomyosarcoma, leiomyosarcoma

## Abstract

Inflammatory leiomyosarcoma (ILMS) is a rare malignant soft tissue neoplasm with smooth muscle differentiation, prominent inflammatory infiltration, and near-haploidization. It is extremely rare in the head and neck region, and no intraoral cases have been reported.

The lesion was initially diagnosed as a malignant spindle cell neoplasm at the referring laboratory. Microscopic examination of blocks of excised fragmented lesion revealed a cellular neoplasm composed of plump, spindle-shaped cells with blunt-ended and elongated nuclei and eosinophilic fibrillary cytoplasm arranged in a fascicular, herringbone to haphazard pattern. The tumor cells were interspersed with mixed inflammatory infiltration and were diffusely positive to desmin, SMA, H Caldesmon, and MYOD1. The diagnosis came as Inflammatory leiomyosarcoma.

This case is the first reported case of ILMS involving the oral cavity. Even though this lesion is very rare, this neoplasm should be included in the differential diagnosis of a spindle cell lesion with marked lymphohistiocytic infiltration.

## Introduction

Inflammatory leiomyosarcoma is a rare, malignant soft tissue neoplasm with smooth muscle differentiation, prominent inflammatory infiltration, and near-haploidization; it was first recognized by Merchant et al. in 1995 and was recognized by the WHO as a distinct entity in 2020 [1,2]. Clinically, ILMS mainly occurs in adults with a male predilection. Most ILMS cases presented as an enlarged indolent soft tissue mass with no specific feature; however, inflammatory symptoms had been reported in some cases [3-5].

ILMS mostly involves the deep soft tissue of the lower limb, trunk, and retroperitoneum area and rarely involves the lung. ILMS is extremely rare in the head and neck region, with only a few reported cases in the literature [5-8].

Histopathologically, the tumor showed an eosinophilic spindle cell arranged in a fascicle, storiform, or haphazard pattern with variable degrees of pleomorphism, atypia, and mitosis with prominent inflammatory infiltration, including lymphocytes, xanthoma cells, and infrequently neutrophils or eosinophils. Immunohistochemistry of such lesions shows co-expression of smooth and skeletal muscle markers [5,7].

Surgical management with a clear margin is the favored treatment of such lesions. Furthermore, adjuvant chemotherapy and radiotherapy have been reported [6,8]. Due to the rarity of this tumor, there is a limited follow-up date with an overall good prognosis. Metastasis has been reported in a few cases [3,6].

To our knowledge, no intraoral ILMS has been reported in the literature. Here, we report a case of ILMS involving the buccal mucosa of a 30-year-old Saudi male patient and discuss the differential diagnosis of this rare lesion.

## Case presentation

A 30-year-old male patient presented with the chief complaint of swelling on the left side of the face. The patient reported that the lesion appeared approximately one year ago and was painless except when he bit it, and it seemed to increase in size in the last four months. The patient's medical history was unremarkable. Laboratory tests were within normal limits, including complete blood count, coagulation profile, and renal and hepatic profile. In addition, the HIV test was negative.

**Figure 1:**
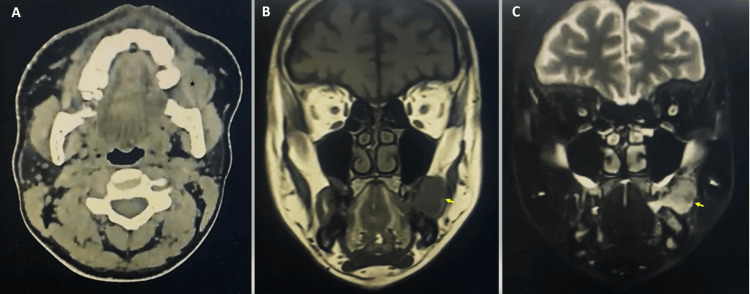
Radiographic imaging A) CT scan showed a soft tissue density mass in the left cheek (asterisk). B) MRI showed a well-circumscribed mass, hypointense in T1. C) Iso to hyperintense mass in T2 (arrows).

The extraoral examination showed facial asymmetry and swelling of the left cheek with normal overlying skin. The intraoral examination revealed a painless, dome-shaped exophytic sessile lesion with ulcerative surface, where the patient bit the mass, located on the buccal mucosal. It was firm to rubbery in consistency. A CT scan radiograph revealed a homogenous soft tissue density mass extending laterally to the left cheek's subcutaneous plane and medially to the oral cavity. MRI radiographs showed a well-defined, soft tissue neoplasm that was hypointense in T1 and iso to hyperintense in T2 with evidence of contrast enhancement (Figures 1A-1C).

The lesion measured about 4.2 X 3 cm. The lesion was excised under general anesthesia.

At our institute, we reviewed blocks of a fragmented, firm lesion that appeared grossly grayish to tan. The lesion was initially diagnosed as a malignant spindle cell neoplasm at the referring laboratory.

**Figure 2:**
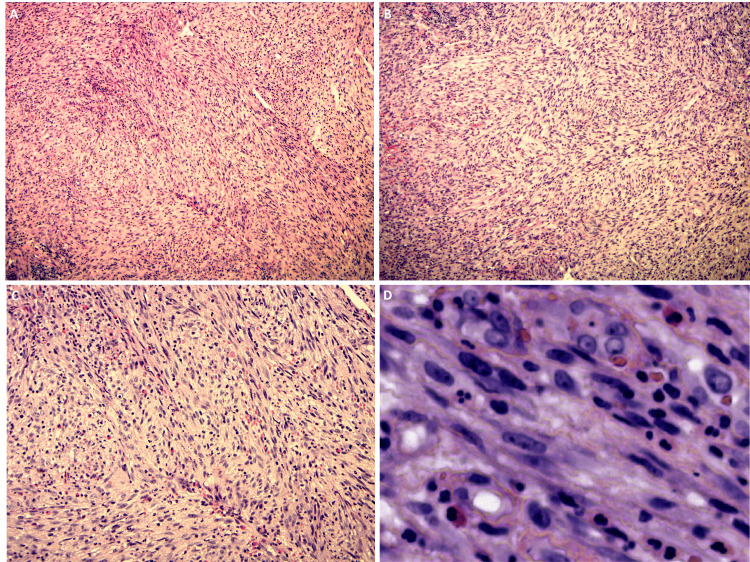
The tumor's microscopic characteristics At low magnification (A and B), the tumor cells are arranged in a fascicular, herringbone, and haphazard pattern. Higher magnification (C) reveals a notable mixed inflammatory infiltration, including lymphocytes, histocytes, and eosinophils. The tumor cells exhibit characteristic features at the highest magnification (D) Spindle-shaped cells with elongated and blunt-ended nuclei.

Microscopic examination revealed a cellular neoplasm composed of plump, spindle-shaped cells with blunt-ended and elongated nuclei and eosinophilic fibrillary cytoplasm. The tumor cells are arranged in a fascicular, herringbone, to haphazard pattern in a fibrovascular stroma. The tumor cells were interspersed with mixed inflammatory infiltration, including histocytes and lymphocytes. In addition, the tumor cells showed minimal pleomorphism and hyperchromatism with numerous mitoses and a lack of necrosis (Figures 2A-2D).

**Figure 3:**
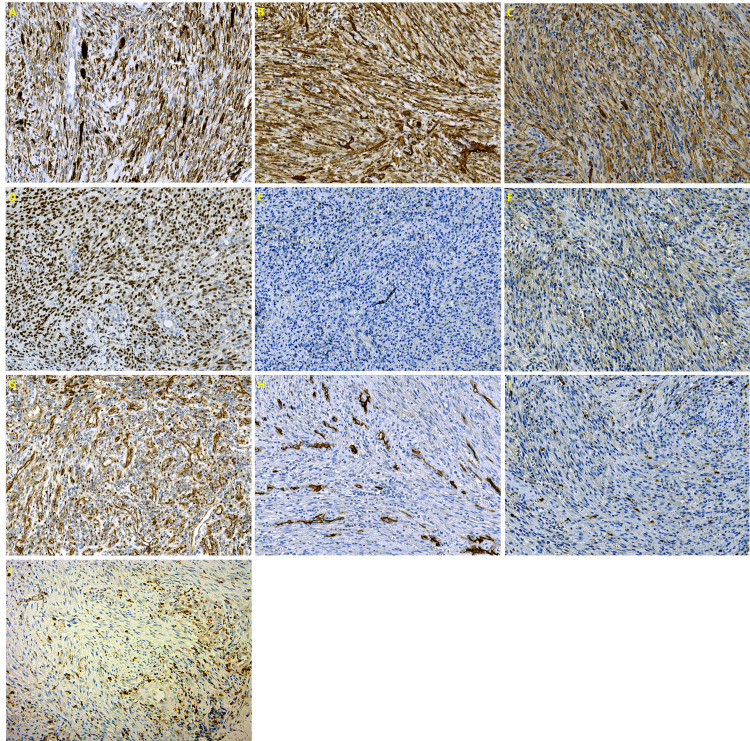
Immunohistochemical stain results The tumor cells are (A) Diffusely positive for desmin, (B) Diffusely positive for SMA, (C) Diffusely positive for H Caldesmon, (D) Diffusely positive for MyoD1, (E) Negative for myogenin, (F) Negative for ALK, (G) cytoplasmic positivity for β-Catenin, (H) Negative for CD34, and (I) Negative for S100; (J) CD68 highlighting the interspersed histocytes.

Immunohistochemically, the neoplasm cells were diffusely positive to desmin (DE-R-11, Ventana), smooth muscle actin (alpha sm-1, Leica, Wetzlar, Germany), H Caldesmon (E89, Cell Marque, Rocklin, CA, US), and MyoD1 (EP212, Cell Marque), and cytoplasmic positivity to β-Catenin (β-catenin-1, Dako, Glostrup, Denmark) while negative to S100 (4C4.9, Ventana, Arizona, US), CD34 (QBEnd /10, Ventana ), factor XIIIa (E980.1, Leica), ALK (5A4, Leica), myogenin (F50; Dako), and EBER (CS.1-4, Dako). Moreover, immunohistochemical staining with CD68 (514H12, Leica) revealed a significant presence of interspersed histocytes (Figures 3A-3J).

Based on the histopathologic examination and immunohistochemical staining, the diagnosis was inflammatory leiomyosarcoma. The patient was followed for 15 months with no evidence of recurrence.

## Discussion

Merchant et al. were the first to identify ILMS in 1995. They reported a case series of 12 tumors displaying morphological and immunohistochemical characteristics that resembled smooth muscle differentiation and exhibited a significant inflammatory component. These tumors were previously categorized as inflammatory malignant fibrous histiocytoma [1]. ILMS recently has been designated as a separate entity in the last WHO Classification of Tumors (fifth edition) under the category of smooth muscle tumor [2]. It mostly occurs in adults with male predilection and mainly involves the deep soft tissue of the lower limb, trunk, and retroperitoneum area and rarely involves the lung [6,7]. ILMS is extremely rare in the head and neck region, with only three reported cases in the literature. One case was written by Viljoen N et al. as an asymptomatic lump involving the right lower neck, another by Cloutier JM et al. as a histiocyte-rich rhabdomyoblastic tumor involving the parapharyngeal soft tissue, and a third by Rekhi B et al. as a low-grade inflammatory leiomyosarcoma/histiocyte-rich rhabdomyoblastic tumor involved the back of the neck [5,7,8]. The clinicopathologic characteristics of those cases, including our case, are summarized in Table 1.

**Table 1:** Clinicopathological and immunohistochemical features of inflammatory leiomyosarcomas involving the head and neck region previously published in the literature, including our case

Serial No.	Study	age	site	clinical	radiograph	IHC	genetic	treatment	Follow up
1	Our case	A 30-year-old male	One-year history of asymptomatic left fascial swelling, increase in size in the last four months.	Painless, rubbery to firm, dome-shaped exophytic sessile lesion with ulcerative surface, where the patient bit the mass, located on the left buccal mucosal and measured 4.2 X 3 cm.	CT scan: homogenous soft tissue density mass extending laterally to the left cheek's subcutaneous plane and medially to the oral cavity. MRI: a well-defined soft tissue neoplasm that was hypointense in T1 and iso to hyperintense in T2 with evidence of contrast enhancement.	+ve: desmin, SMA, MYOD1, and H Caldesmon. -ve: S100, CD34, ALK1, FXIIIa, B catenin, Myogenin, EBER, and CD21.	Not performed	Wide local excision	15 months without recurrence
2	Viljoen N [5].	A 37-year-old male	Four-year history of an asymptomatic lump in the right lower neck	Hard mass in the inferior third of the right sternocleidomastoid muscle.	Well-defined homogenously enhancing mass in the right supraclavicular region at the inferior level of the thyroid gland.	+ve: desmin and SMA (diffuse), Myogenin (Focal). -ve: S100, AE1/AE3, ALK1, EBER.	Not mentioned	Wide local excision	Not mentioned
3	Cloutier JM [7]	A 4-year-old female	Parapharyngeal soft tissue	Not mentioned.	Not mentioned.	+ve: desmin, SMA Myogenin, MYOD1, and PAX7. -ve: H Caldesmon.	Near-haploid by SNP array	Wide local excision	No (recent case)
4	Rekhi B [8]	A 17-year-old male	Back of the neck of one-year duration, which seemed to be increasing in size over the last 6 months	A firm, immobile lump over the right side of the neck behind the mastoid area was noted, measuring 5 cm x 4 cm.	Well-defined, hypodense lesion in the intermuscular plane over the postero-inferior and lateral aspect of the occipital region, extending up to the right mastoid region.	+ve: desmin and MYOD1 (diffuse), SMA, and Myogenin (Focal). -ve: S100, SOX10, H Caldesmon, ALK, retain INI1.	Negative for MYOD1 (L122R) mutation (PCR test)	Surgical excision and radiotherapy	Free of disease for 12 months

The histopathological examination of the current case revealed the presence of spindle cells, which prompted consideration of several potential differential diagnoses, including EBV-associated smooth muscle tumor (EBV-SMT), inflammatory myofibroblastic tumor (IMFT), spindle cell rhabdomyosarcoma, follicular dendritic cell sarcoma, fibrous histiocytoma, desmoid fibromatosis and malignant peripheral nerve sheath tumor (MPNST).

EBV-SMT is only seen in immunosuppressive patients in a setting of HIV/AIDS after transplantation and congenital immunodeficiency. It is characterized by smooth muscle differentiation and EBV positivity. In the presented case, the patient was immunocompetent and was negative for EBV transcript by in situ hybridization [9,10].

IMFT is a mesenchymal neoplasm with spindle fibroblastic-myofibroblastic cells and mixed inflammatory infiltration with dominant lymphoplasmacytic infiltration. The immunohistochemical profile for IMFT shows variable staining for SMA, MSA, and desmin; the nuclear positivity to ALK is observed in approximately 50-60% of cases, demonstrating a strong correlation with ALK gene rearrangement. In our case, the positivity to MYOD1 and Caldesmon and negativity to Alk exclude the diagnosis [2,11].

Spindle cell rhabdomyosarcoma is commonly found in the head and neck area and usually arranged in storiform and intersecting fascicles of spindle cells with infiltrative growth and prominent nuclear atypia and mitotic figure. The well-circumscribed appearance of our lesion with prominent inflammatory infiltration and foamy histocyte, in addition to positivity to h-Caldesmon, helps to differentiate both lesions [2,6,12].

Extranodal follicular dendritic cell sarcoma is an extremely rare tumor that can affect the oral cavity and may easily be misdiagnosed. It is prone to be confused with ILMS in histopathology, as it is a well-circumscribed lesion that shows oval to spindle cells arranged in fascicular, storiform, and whorled growth patterns with lymphohistiocytic infiltration. The positivity to desmin and the negativity to dendritic cell markers like CD21, CD23, and CD35 in the presented neoplasm rule out this diagnosis [13,14]. The other differential diagnoses were excluded, as our lesion was positive to H-Caldesmon and MYOD1 and negative to FXIIIa, B-catenin, and S100.

H-Caldesmon is recognized as the most specific marker to distinguish smooth muscle differentiation [7,15,16]. Cloutier JM et al. stated in their paper, “We are not aware of any cases of ILMS or HRRMT which have been shown to be Caldesmon-positive, using the h-CD clone”. However, in our case, the lesion was diffusely positive to H-Caldesmon [7].

At the molecular level, ILMS is characterized by near-haploid loss of heterozygosity in most chromosomes but consistently maintained heterozygosity for chromosomes 5 and 22 and frequently for chromosomes 18, 20, and 21. In addition, NF1 and TP53 gene mutations have been reported [2,8,17]. Regrettably, the genetic test was not done in our case. Immunohistochemistry remains essential for accurate diagnosis because cytogenetic analysis is not technically achievable for most laboratories.

The quest for the best terminology for ILMS still exists. Cloutier JM et al. proposed the name “inflammatory rhabdomyoblastic tumors” and Michal et al. proposed the term “low-grade inflammatory myogenic tumor” based on the indolent clinical behavior of the lesion and interrogation of the smooth muscle lineage [6,7].

## Conclusions

This case report presented the first reported case of ILMS involving the oral cavity. Even though this lesion is very rare, this neoplasm should be included in the differential diagnosis of a spindle cell lesion with marked lymphohistiocytic infiltration, as this can protect the patient from receiving aggressive, unnecessary treatment.
